# Glomerular Filtration Rate (GFR) determination via individual kinetics of the inulin-like polyfructosan sinistrin versus creatinine-based population-derived regression formulae

**DOI:** 10.1186/1471-2369-14-159

**Published:** 2013-07-22

**Authors:** Sabine Zitta, Walter Schrabmair, Gilbert Reibnegger, Andreas Meinitzer, Doris Wagner, Willibald Estelberger, Alexander R Rosenkranz

**Affiliations:** 1 Department of Internal Medicine, Clinical Division of Nephrology, Medical University Graz, Graz, Austria; 2 Institute of Physiological Chemistry, Center for Physiological Medicine, Medical University Graz, Graz, Austria; 3 Department of Laboratory Medicine, Medical University Graz, Graz, Austria; 4 Department of Surgery, Division of Transplantation, Medical University Graz, Graz, Austria

**Keywords:** eGFR, MDRD, CKD-EPI, mGFR, Sinistrin, Kinetics

## Abstract

**Background:**

In renal patients estimation of GFR is routinely done by means of population-based formulae using serum creatinine levels. For GFR determination in the creatinine-blind regions or in cases of reno-hepatic syndrome as well as in critical cases of live kidney donors individualized measurements of GFR (mGFR) employing the kinetics of exogenous filtration markers such as the inulin-like polyfructosan sinistrin are necessary. The goal of this study is to compare mGFR values with the eGFR values gained by the Modification of Diet in Renal Disease (MDRD4) and Chronic Kidney Disease-Epidemiology Collaboration (CKD-EPI) formulae.

**Methods:**

In 170 subjects comprising persons with normal renal function or with various stages of kidney diseases (CKD 1-4) GFR was measured by application of intravenous bolus of sinistrin and assessment of temporal plasma concentration profiles by means of pharmacokinetic methods (mGFR). Comparisons of mGFR with MDRD4- and CKD-EPI-derived eGFR values were performed by means of linear regression and Bland-Altman analyses.

**Results:**

Reasonable agreement of mGFR and eGFR values was observed in patients with poor renal function [GFR below 60 (ml/min)/1.73 m^2^]. In cases of normal or mildly impaired renal function, GFR determination by MDRD4 or CKD-EPI tends to underestimate GFR. Notably, there is practically no difference between the two eGFR methods.

**Conclusions:**

For routine purposes or for epidemiological studies in cases of poor renal function eGFR methods are generally reliable. But in creatinine-blind ranges [GFR above 60 (ml/min)/1.73 m^2^] eGFR values are unreliable and should be replaced by clinically and physiologically suitable methods for mGFR determination.

**Consort:**

http://www.consort-statement.org/index.aspx?o=1190

## Background

For routine applications or for epidemiological studies, GFR values are estimated on the basis of individual serum creatinine measurements by means of regression formulae, e.g., the MDRD4 and CKD-EPI formulae [1-4]. These so-called eGFR formulae are derived from population-data describing the relations between GFR values obtained by a kinetic methods (mGFR) and the corresponding concentrations of serum creatinine [5].

Unfortunately, these formula-derived GFR-values are problematic in individuals with normal or mildly impaired renal function. Several reasons are responsible for this problem, such as, e.g., the natural variation of the original population data in combination with the confidence intervals increasing with decreasing creatinine concentrations, and the increasing impact of analytical uncertainties of creatinine measurements particularly in low concentration ranges. In addition, disturbed creatinine production, e.g. in patients with liver diseases, may also cause severe limitations for the application of creatinine-based eGFR formulae. Rather, formulae-derived GFR estimations tend to be of use in the presence of moderately or severely impaired renal function, i.e., GFR below 60 (ml/min)/1.73 m^2^ only [6,7].

For accurate assessment of renal function above this threshold pharmacokinetics of exogenous markers such as I^125^-iothalamate or Cr^51^-EDTA or inulin should be used [8]. Sinistrin, an inulin-like polyfructosan, is a physiologically and clinically advantageous GFR marker, which is only filtered by the glomeruli and is metabolically inert [9-12]. In contrast to the original ‘gold standard’ method of constant intravenous infusion of inulin for determination of glomerular filtration rate [13], modern techniques use intravenous bolus injections of test substances and algorithms for fitting of bi-exponential functions to the temporal concentration profiles observed. GFR is determined by dividing the dose applied through the area under the curve estimated with the fitted function parameters [14]. Attainment of a steady state is not required, and only raw guesses of the kinetic constants are required before starting the fitting algorithm.

The aim of the present study is to compare the ranges of validity and the information content which can be gained from the commonly used formulae-based estimations of GFR (MDRD4 and CKD-EPI) with a kinetic technique using sinistrin in a single injection application as standard for comparison, particularly in CKD 1-2. Thereby, the paper emphasizes the use of a kinetic method employing a physiological marker especially suitable for the renal evaluation of live kidney donors or kidney transplant patients. To our knowledge this paper represents the first report on the application of this kinetic procedure in subjects with normal renal function as well as in patients with chronic kidney disease (CKD 1-4).

## Methods

All of the included measurements were performed during routine visits within the standard care program of the Division of Nephrology and Hemodialysis at the Medical University of Graz. An ethical approval was obtained from the ethical committee of the Medical University of Graz to use the results of these examinations for the presented study.

### Subjects

Clearance measurements were performed in 170 subjects (100 females, 70 males), mean age 45 years (range 18.7 – 89.5), mean serum creatinine 1.22 mg/dl (range 0.5 – 6.7), with different underlying diseases: 12 subjects were screened prior to living kidney donation and 36 patients after kidney transplantation. 35 patients suffering from breast cancer were tested prior to chemotherapy, 9 patients with kidney stones prior to lithotripsy, 32 patients had mild hypertension, 14 suffered from diabetes, 21 had glomerulonephritis, 5 patients had a history of unilateral nephrectomy, 5 patients were tested after recompensation of an acute renal failure, and 1 subject had a unilateral duplex kidney. Each subject gave written informed consent. Table 1 shows the distributions of age, body mass and serum creatinine concentration among these groups of patients.

**Table 1:** Basic characteristics of the patients investigated

**Diagnosis**	**Number**	**Age (yr)**	**Body mass (kg)**	**Creatinine (mg/dL)**
Potential live kidney donor	12	45.4 (28.4 – 68.4)	74.4 (59 – 86)	1.0 (0.7 – 1.3)
Kidney transplant recipient	36	43.9 (23.7 – 65.2)	70.1 (50 – 88)	1.5 (0.7 – 3.2)
Malignancy	35	45.3 (23.5 – 89.5)	70.5 (50 – 93)	1.0 (0.5 – 6.7)
Kidney stones	9	43.2 (24.4 -62.9)	85.8 (70 – 104)	1.1 (1.0 – 1.2)
Hypertension	32	48.1 (23.9 – 69.1)	75.3 (51 – 100)	1.1 (0.6 – 2.5)
Diabetes mellitus	14	44.5 (29.0 – 65.9)	73.5 (50 – 95)	1.1 (0.8 – 2.5)
Glomerulonephritis	21	46.2 (22.1 – 74.2)	78.9 (52 – 108)	1.6 (0.5 – 4.1)
Unilateral nephrectomy	5	36.0 (21.8 – 45.2)	69.2 (58 – 86)	1.4 (0.8 – 3.1)
Recompensated acute renal failure	5	44.2 (23.7 – 63.2)	67.4 (55 – 81)	1.0 (0.8 – 1.3)
Unilateral duplexed kidney	1	18.7	58.0	0,8

Shown are mean values and ranges (in parentheses).

### Clinical procedures and techniques of clinical chemistry

Serum creatinine was determined by the Jaffe method (Hitachi, Roche Diagnostic GmbH, Mannheim, Germany) using a kinetic colorimetric assay. The temporal concentration profiles of sinistrin (Inutest®; Fresenius-Kabi, Linz, Austria) after intravenous injection of 2500 mg during 3 minutes were determined by drawing samples from venous blood 10, 20, 30, 45, 60, 90, 120, 150, and 180 min after bolus injection of the marker. After centrifugation of the samples the initial concentration of blood glucose was determined. Thereafter, sinistrin was hydrolyzed to yield fructose monomers. Fructose was enzymatically converted to glucose, and the latter was enzymatically oxidized using NAD. The resulting NADH was assessed photometrically by its extinction of UV [15]. The difference between total and initial glucose concentrations is used to constitute a measure of the sinistrin concentration. All GFR measurements are given standardized in (ml/min)/1.73 m^2^. Within-day CVs for sinistrin were 1.4% at a concentration of 75 mg/L and 1.2% at 275 mg/L. Between-day CVs were 3.3% at 75 mg/mL and 1.6% at 275 mg/L sinistrin.

### Model description and biometric methods

**Figure 1:**
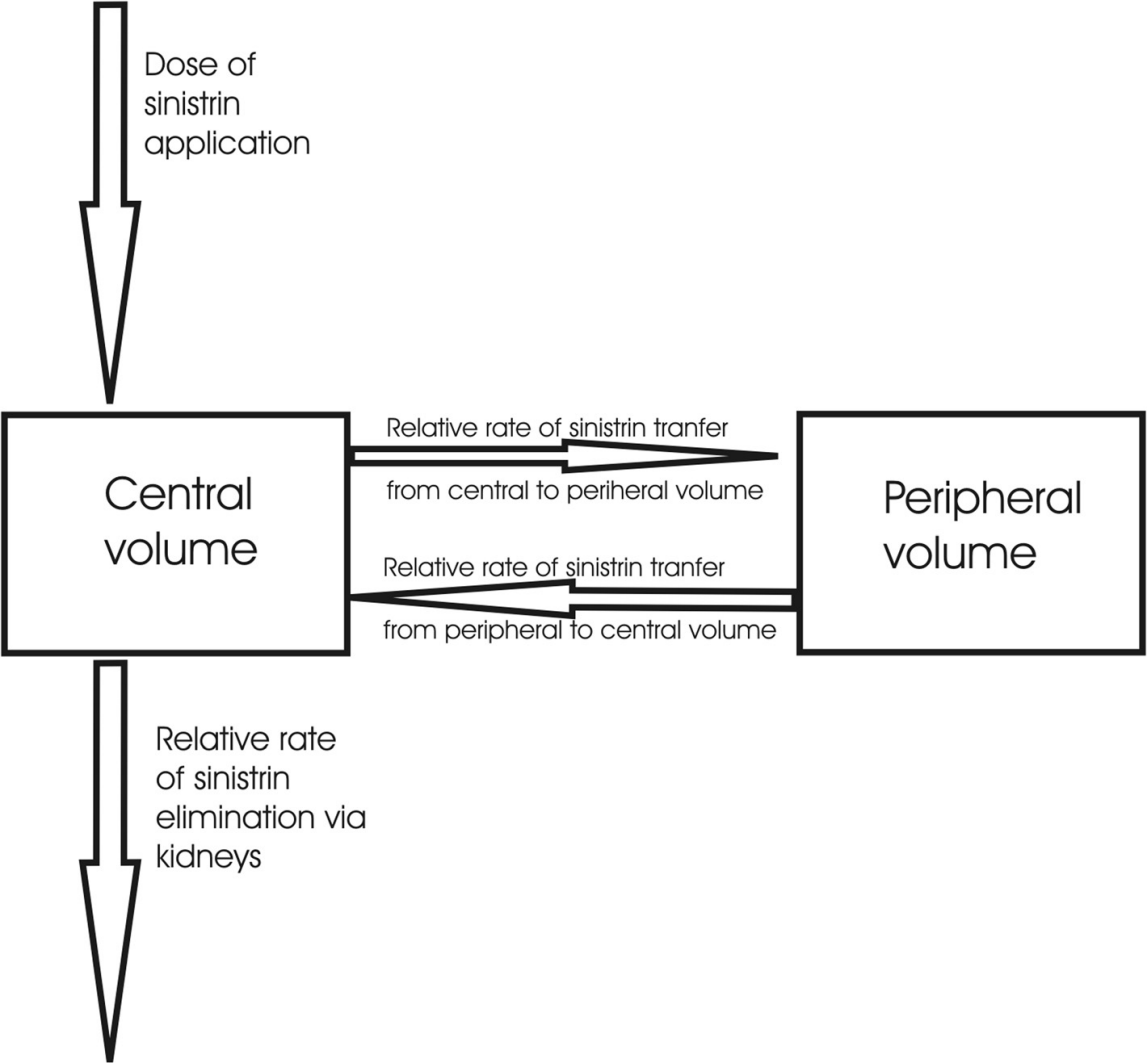
The two-compartment model of pharmacokinetics.

Experiments with sinistrin have shown that its elimination kinetics after a bolus injection can be adequately described by a two-compartment model as depicted in Figure 1[16]. The well-perfused part of the extracellular fluids is considered as the central volume into which the exogenous marker sinistrin is injected, and from which it is on the one hand exchanged with the so-called peripheral compartment comprising the less perfused part of the interstitium, and on the other hand eliminated via the kidneys [17]. Mathematically such a system can be represented by the well-known two-compartment model of pharmacokinetics containing characteristic system constants, namely, the relative transfer rates for the substance exchanges between the two compartments, the rate constant for the eliminating flow from the central compartment to the outside and the volume of the central compartment. From these system constants, or parameters in a mathematical sense, can be derived other model parameters such as the clearance (GFR), the peripheral volume, and a characteristic retention time in the peripheral volume. GFR especially is determined as the product of the rate constant of the elimination and the central volume.

The adaptation of the two-compartment model to the experimentally determined kinetic data profile, yielding the system constants as well as their respective standard estimation errors due to the noise in the experimental data, was done by embedding the analytical solution of the model [18] into a nonlinear regression procedure [19]. However, it could also be done by employing a commercially available pharmacokinetic software package (SAAM II, Software Application for Kinetic Analyses, Version 2.0, ©University of Washington) [20].

GFR estimations based on serum creatinine were performed by means of the MDRD4 – and CKD-EPI formulae [21]. The abbreviated four-variable MDRD eGFR was calculated as follows:

eGFR (mL/min) = 186 × SCr ^-1.154^ × age ^-0.203^ (× 0.742 if female) (× 1.21 if black)

The CKD-EPI GFR was calculated gender specifically and stratified by creatinine levels according to the following equations:

Female with SCr ≤ 0.7 mg/dl

eGFR (ml/min) = 144 × 0.993 ^age^ × (SCr / 0.7) ^-0.329^ (× 1.15 if black)

Female with SCr > 0.7 mg/dl

eGFR (ml/min) = 144 × 0.993 ^age^ × (SCr / 0.7) ^-1.209^ (× 1.15 if black)

Male with SCr ≤ 0.9 mg/dl

eGFR (ml/min) = 141 × 0.993 ^age^ × (SCr / 0.9) ^-0.411^ (× 1.16 if black)

Male with SCr > 0.9 mg/dl

eGFR (ml/min) = 141 × 0.993 ^age^ × (SCr / 0.9) ^-1.209^ (× 1.16 if black)

An initial comparison of kinetically measured mGFR with creatinine-based eGFR values was performed by linear regression and correlation techniques. It is well known, however, that these techniques are not well suited for assessing agreement between different analytical methods, and thus, we also analyzed our data according to the method by Bland and Altman [22,23]: the differences between the two methods (bias = mGFR – eGFR) are plotted against the common mean values. Thus, the bias of the two methods, together with its 95% confidence intervals and the so called limits of agreement can be obtained.

## Results

**Figure 2:**
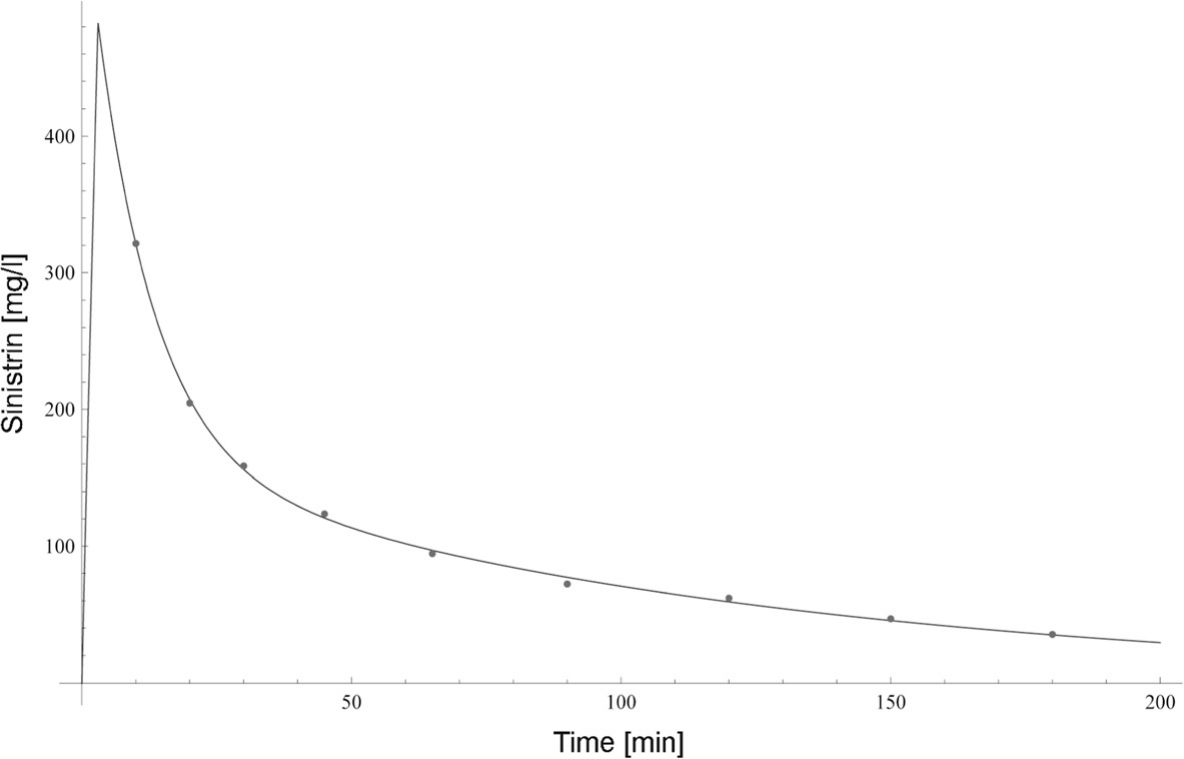
Temporal profiles of observed (small circles) and model-adapted concentration of sinistrin (smooth line) after an intravenous bolus of 2500 mg sinistrin in a normal subject.

In order to illustrate the method chosen for the measurement of GFR, the result of fitting a pharmacokinetic two-compartment model to the observed temporal concentration profile of sinistrin is given for an individual with normal renal function (Figure 2).

**Figure 3:**
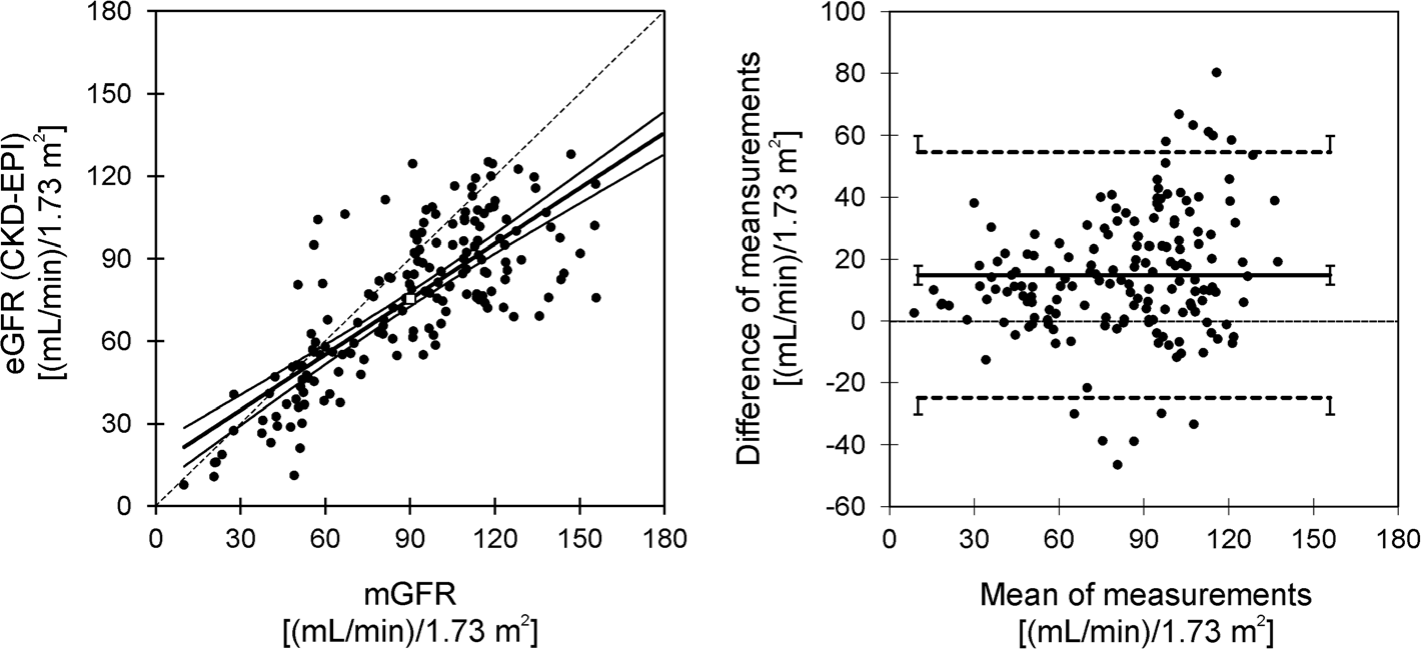
**Analysis of agreement between mGFR and eGFR according to the CKD-EPI formula. Left**: Linear regression analysis results: the thick line represents the regression line; the two medium thick hyperbolic lines show the 95% confidence intervals of the regression line. For comparison, the thin dashed line along the diagonal represents the line of identity. **Right**: Results of the Bland-Altman analysis: the solid horizontal line shows the bias, and the two dashed horizontal lines visualize the limits of agreement between both methods. For all three lines, also the 95% confidence intervals are indicated. Note that bias is defined as mean of mGFR minus mean of eGFR.

To illustrate the association between kinetically determined GFR and creatinine based eGFR, Figure 3 shows the results obtained for the CKD-EPI formula: the left panel of Figure 3 shows a classical linear regression analysis, and the right panel visualizes the outcome of the Bland-Altman technique for the same data. (The MDRD4 formula would yield a graphical representation being nearly indistinguishable from Figure 3; so we do not show it explicitly.) Obviously, while the linear regression apparently shows reasonably narrow 95% confidence bounds of the regression line, there is considerable scatter of the measurements and, more importantly, the slope of the regression line deviates significantly from the line of identity (which would indicate perfect agreement of the two methods). Rather, the slope indicates significant underestimation of eGFR values, compared with the kinetically determined mGFR. In agreement with these unfavorable conclusions from the linear regression analysis, also the Bland-Altman analysis shows statistically highly significant positive bias (the eGFR values are consistently lower than mGFR), and the limits of agreement between the two methods indicate inacceptable deviations.

Table 2 (column “Total mGFR range”) substantiates these findings in quantitative manner for both eGFR methods. As the Bland-Altman plot in Figure 3 indicates, however, the deviations between the kinetically obtained mGFR and either of the eGFR tend to grow with growing mean value of the measurements: the bias obviously is smaller for small mean values, and larger for large mean values. Therefore, Table 2 shows also the results obtained by stratifying the statistical analyses according to mGFR: including into the analyses only patients with severely disturbed renal function [mGFR <= 60 (mL/min)/ 1.73 m^2^], the statistical criteria show much better agreement between mGFR and both eGFR values; however, patients with better renal function [mGFR > 60 (mL/min)/ 1.73 m^2^] in accordance with the graphical representation exhibit even worse results. Splitting the latter group of patients again according to a cut-off threshold of mGFR smaller or larger than 90 (mL/min)/ 1.73 m^2^ (mildly disturbed versus normal renal function), similar results are obtained with the worst agreement seen in individuals with normal mGFR > 90 (mL/min)/ 1.73 m^2^. (Further stratification was not performed since in the smaller strata the number of individuals becomes critically low.)

**Table 2:** Results of the linear regression analyses as well the Bland-Altman-analyses of the creatinine-based eGFR values (dependent variable) versus the kinetically determined mGFR values (95% confidence intervals in parentheses)

**eGFR Variable**		**Total mGFR range**	**mGFR <= 60 (mL/min)/1.73 m**^**2**^	**mGFR > 60 (mL/min)/1.73 m**^**2**^	**60 < mGFR <= 90 (mL/min)/1.73 m**^**2**^	**mGFR > 90 (mL/min)/1.73 m**^**2**^
**CKD-EPI**	Intercept	14.7 (6.9 – 22.4)	−9.8 (-29.5 – 9.9)	34.7 (20.5 – 48.9)	1.7 (-44.8 – 48.2)	64.9 (41.3 – 88.2)
	Slope	0.67 (0.59 – 0.75)	1.13 (0.71 – 1.55)	0.49 (0.36 – 0.62)	0.86 (0.25 – 1.47)	0.24 (0.04 – 0.44)
	R*	0.78 (0.71 – 0.83)	0.66 (0.44 – 0.80)	0.55 (0.42 – 0.66)	0.47 (0.14 – 0.71)	0.23 (0.03 – 0.41)
	Bias^+^	14.8 (11.8 – 17.9)	3.8 (-1.3 – 8.9)	18.4 (14.8 – 21.9)	8.6 (3.2 – 14.1)	21.5 (17.3 – 25.7)
	Limits of agreement	−24.9 – 54.6	−27.9 – 35.6	−21.4 – 51.9)	−20.2 – 37.6	−19.3 – 62.2
						
**MDRD**	Intercept	14.9 (7.0 – 22.9)	−6.7 (-24.4 – 11.0)	34.7 (19.0 – 49.1)	10.4 (-30.2 – 51.1)	61.3 (35.4 – 87.2)
	Slope	0.64 (0.55 – 0.72)	1.03 (0.66 – 1.41)	0.46 (0.32 – 0.60)	0.72 (0.18 – 1.25)	0.24 (0.01 – 0.46)
	R	0.76 (0.69 – 0.82)	0.67 (0.46 – 0.81)	0.50 (0.36 – 0.62)	0.45 (0.11 – 0.69)	0.21 (0.01 – 0.3)
	Bias	17.9 (14.7 – 21.1)	5.2 (0.6 – 9.7)	22.0 (18.2 – 25.7)	11.1 (6.3 – 15.9)	25.4 (20.9 – 29.9)
	Limits of agreement	−23.5 – 59.3	−23.1 – 33.5	−19.9 – 63.9	−14.6 – 36.9	−18.4 – 69.3

*R, linear correlation coefficient.

^+^Bias is defined as mean of mGFR minus mean of eGFR.

There is no real difference between the MDRD4- and the CKD-EPI-methods over the GFR ranges studied. As can be seen from Table 2, the results of the linear regression as well as the Bland-Altman analyses using either MDRD or CKD-EPI-eGFR values as dependent variable are nearly identical, both over the total GFR range and in the various subranges studied. As expected, therefore, a linear regression analysis between MDRD and CKD-EPI results yields a nearly perfect agreement with an intercept of 0.24 (95% c.i., -2.0 – 2.5), a slope of 0.96 (0.93 – 0.98) and a correlation coefficient of 0.98 (0.976 – 0.987). The Bland-Altman analysis of the agreement between both eGFR measurements yields a bias of 3.1 (2.3 – 3.9) with acceptable limits of agreement (-7.1 – 13.3). Accordingly, there are also no relevant differences in the overall diagnostic powers of the two eGFR formulae, taking 60 ml/min/1.73 m^2^ as mGFR cut-off value gained by the kinetic procedure: for the MDRD-formula the diagnostic specificity is 0.875, whereas for the CKD-EPI-formula it is 0.891. The diagnostic sensitivity for the MDRD-formula is 0.902, whereas for the CKD-EPI-formula it is 0.878.

## Discussion

The purpose of the present study is to compare the application of compartment analysis techniques using kinetic data of the GFR marker sinistrin with formula-derived GFR estimates based on creatinine levels. The kinetic procedure used here as a standard for comparison, has been validated previously by successfully predicting in individual patients the concentration-profile of a constant infusion experiment using results obtained by a bolus experiment beforehand [18]. The model-derived mGFR values were used to assess the reliability of eGFR estimates obtained by the MDRD4- and CKD-EPI-formulae in individuals with poor renal function as well as in subjects with normal or only mildly disturbed kidney function. (Of all existing MDRD formulae, we chose the MDRD4 equation as is was the most common one at the time when this study was started. Therefore, we used the earlier factor 186 instead of the later established value 175.) Similar problems have been studied in the literature [24,25]. A weak point of the present study is that a retrospective data base arising from everyday clinical routine had to be used. However, similar results as in Figure 3 were presented previously using mGFR values gained by constant infusion of inulin versus creatinine based eGFR values [26].

As Table 2 shows, in patients with mGFR values indicating severely disturbed renal function and, thus, high concentrations of serum creatinine, eGFR methods may suffice despite many objections against them [27-30]: while the linear correlation coefficient still is far from perfect in this range of renal function, the slope of the regression line is not significantly different from unity, and, even more important, the bias according to the Bland-Altman technique is comparatively low. The necessity for methods more exact than the simple creatinine-based estimations, however, arises in the ranges of only moderately restricted or even normal renal function. In fact, this deficit of creatinine-based estimations is not really a surprise taking into account the flatness of the relationship between serum creatinine concentrations and GFR values in the range of normal or only mildly disturbed renal function [31,32]. Besides the inherent weakness of eGFR values in the creatinine-blind ranges of low creatinine levels associated with laboratory errors, physiological aspects are also to be considered in judging renal function. Especially individual variations in distribution volumes are not taken into account by eGFR formulae.

One might ask whether it is necessary to perform a full kinetic analysis of the time-dependent concentration curves of a marker like sinistrin or whether a simple analysis of the area under the concentration curve would suffice. There are several arguments supporting the full kinetic analysis: first, considering the relevant large volumes of marker solutions and the individually different states of the veins of the patients, the administration of the marker during a reasonable time – 3 minutes instead of a truly instantaneous bolus injection – is possible. Moreover, it is practically impossible to employ a too extended experimental protocol. Both these practical limitations are in favor of the full kinetic analysis of the applied marker. Second, the availability of sufficient computer power enables one to extract more information out of concentration curves of exogenous markers. This is of importance in critical cases as, e.g., in live kidney donors. Third, by means of the full kinetic analysis it is possible to approach much more complicated scenarios: for example, the immediate response of the kidneys to external dietetic or pharmacologic loads, which may cause both changes in GFRs as well as shifts in the extracellular fluids from central to peripheral compartments, can be studied with the same method, yielding information on the state of the renal vasculature. For this purpose it is necessary to perform two consecutive experiments with the external loads in between. In the second experiment, this procedure requires the calculation of the remaining marker amounts in the two compartments, since now these remaining amounts are needed as initial values for the model identification describing the second experiment. Moreover, the kinetic techniques allow also the determination of the error bounds of the characteristic system constants [33]. The application of an exogenous marker such as sinistrin and the appropriate model-identification of the kinetics involved enables one to determine the individual mGFR and measures of the distribution process such as the extracellular volume in the individual subject [34]. For a critical judgment of the measured values in individual patients reliable error measures of all these various pharmacokinetic parameters are indispensable; and they can be gained only by a model-fitting procedure. So while in the present paper we have concentrated only on the relationships between mGFR and the eGFR variables, we nevertheless strongly advocate a full kinetic analysis because of its far-reaching potential.

Sinistrin is a clinically and physiologically suitable marker and the bolus method assessed by compartmental kinetic analysis is a both reliable and clinically practicable procedure for the measurement of GFR. This is of utmost importance in subjects requiring higher than routine accuracy and precision as, e.g., in situations of live kidney donor evaluations [35]. As stated in the Amsterdam consensus guidelines [36] kidneys from live donors with a GFR < 80 ml/min are associated with relative risk of graft loss of 2.28 compared to those with higher GFR prior to nephrectomy. In this context GFR measurement applying sinistrin kinetics appears to be of particular use for exact determination of GFR in subjects with normal or mildly impaired renal function. In subjects with normal or close to normal renal function commonly used GFR equations based on serum creatinine are unreliable and thus should be avoided for the evaluation of live kidney donors.

## Conclusion

For correct determination of GFR, particularly in subjects with normal or slightly impaired renal function (CKD 1-2), kinetic procedures using an exogenous physiologically and clinically suitable marker such as sinistrin allow individualized GFR measurements in contrast to population-derived estimations by means of formulae employing endogenous markers. Additionally, a model-based technique enables one to assess consecutive clearance tests with a pharmacological or dietetic load in between. Such tests can be used for obtaining information on the properties of the renal functional reserve and the renal micro-vasculature, e.g., on changes in its permeability caused by renal injuries [37]. Dynamic renal function testing of this sort is not possible by means of endogenous markers [38].

Summarizing it can be stated that both the accuracy and precision of mGFR values achievable by the kinetic procedure using sinistrin as exogenous marker as well as the possibility for evaluation of the renal functional reserve appear to be of decisive importance in cases of live kidney donors. Finally although so far eGFR methods using creatinine levels only have been considered as sufficiently satisfactory, recent developments suggest combinations of creatinine and cystatin C in regression formulae as necessary improvements in GFR estimations [39]. However, any such progress still suffers from the drawback of employing statistical formulae derived from population data based on endogenous markers and not from individual kinetic measurements based on exogenous markers.

## Abbreviations

CKD-EPI: Chronic Kidney Disease – Epidemiology Collaboration; MDRD: Modification of Diet in Renal Disease; eGFR: Glomerular filtration rate estimated on basis of serum creatinine concentration; mGFR: Glomerular filtration rate measured by full kinetic analysis.

## Competing interests

The authors declare that they have no competing interests.

## Authors’ contributions

*SZ:* Made substantial contributions to conception and design and performed GFR measurements. *WS, GR and WE:* Performed statistical analyses, contributed to interpretation of data, and revised the manuscript critically for important intellectual content. *AM*: Performed analytical procedures. *DW:* Performed GFR measurements. *ARR:* Made substantial contributions in drafting the manuscript and revised it critically for important intellectual content. The final version of this paper was approved by all authors.

## Pre-publication history

The pre-publication history for this paper can be accessed here:

http://www.biomedcentral.com/1471-2369/14/159/prepub
